# Intraoperative Chemosis During Resection of Lower Eyelid Lesion

**Published:** 2015-09-16

**Authors:** Jack Burns, Joshua B. Elston, Michael A. Harrington

**Affiliations:** ^a^Division of Plastic Surgery, Department of Surgery, University of Kentucky College of Medicine, Lexington; ^b^Division of Plastic Surgery, Department of Surgery, University of South Florida Morsani College of Medicine, Tampa

**Keywords:** chemosis, eyelid reconstruction, cantholysis, Tenon's capsule, dellen formation

## DESCRIPTION

A 47-year-old woman presented for surgical resection of basal cell carcinoma of the right lower eyelid with immediate reconstruction. Resection and reconstruction were successfully performed; however, intraoperatively, the patient rapidly developed profound chemosis.

## QUESTIONS

1. **What is chemosis and what are some predisposing factors?**
2. **What is the pathogenesis of chemosis?**
3. **What is the preferred management of chemosis?**
4. **What surgical techniques may be used to minimize the risk for developing chemosis?**

## DISCUSSION

Chemosis is defined as edema of the conjunctiva. It is most commonly seen immediately following surgical procedures on the eye, especially the lower eyelid, but may arise any time during the postoperative period.^1^ The severity of chemosis can vary greatly and is usually categorized by the degree of prolapse between the eyelids. Mild chemosis is described as mild conjunctival protrusion, whereas severe chemosis is seen when there is an impairment of eyelid closure.^2^

Chemosis is commonly caused by impaired lymphatic drainage, increased inflammation, and/or venous congestion. Allergic reaction, infection, or trauma may be the precipitating event that induces an inflammatory conjunctival response. The course of chemosis is thought to be caused by a positive feedback loop: conjunctival swelling leads to desiccation and increased inflammation. As the swelling increases, there is worsening malposition of the eyelid and the cornea, resulting in a disruption in tear flow dynamics over the surface of the eye.^2^^,^^3^ The cornea has a dehydrating mechanism that helps maintain transparency and is based on tear flow in the limbus. With tear flow changes, the dehydrating mechanism is overactivated, leading to thinning of the cornea and loss of corneal epithelium, a process commonly referred to as dellen formation.^2^ Dellen formation leads to increased local inflammation, thus restarting the positive feedback loop.

The treatment of chemosis is based on blocking or disrupting the positive feedback loop. Severe intraoperative chemosis is treated with a conjunctival and Tenon's capsule incision to release fluid from the subconjunctival space.^4^ Initial treatment of mild and chronic postoperative chemosis is with phenylephrine 2.5% drops, dexamethasone 0.1% drops, and lubricating drops. If no resolution is seen with this regimen, the eye is often patched for 24 hours, with firm pressure on the eye to reduce swelling.^2^^,^^5^ Initiation of systemic anti-inflammatories in addition to the pressure eye patch can be considered in severe cases. With chronic chemosis, there may be persistent swelling in the absence of inflamed tissue. In these cases, it may be necessary to incise or excise the affected conjunctiva and Tenon's capsule.^5^ Finally, many instances of prolonged chemosis are caused by mechanical dysfunction of the eyelids related to lid malposition, which requires surgical correction.^2^

In procedures such as cantholyses or canthotomies, care should be taken to avoid the lymphatic vessels lateral to the eye and surrounding the cheek. Both veins and lymphatic vessels course under the superficial musculoaponeurotic system (SMAS) in the cheek region. Oftentimes, dissection in this area is carried out in the sub-SMAS layer, leading to destruction of the lymphatic vessels and an increased risk of chemosis. Alternatively, a dissection under the periosteum avoids significant trauma to the sub-SMAS layer and markedly reduces the risk of chemosis.^6^

In the case described earlier, the patient developed chemosis intraoperatively, which is rarely seen.^5^ Typically, chemosis will develop several hours to days following eye procedures. The etiology of this patient's chemosis was not clearly determined but was believed to be multifactorial. Possible etiologies include an allergic reaction from the eye lubricant used to insert a corneal protector, irritation from the corneal protector on the globe, and/or dissipating thermal energy to the cornea from nearby use of electrocautery. In this case, the patient was treated with phenylephrine drops, steroid drops, and frequent lubricating drops. She was followed closely for several weeks after her procedure and by 4 weeks postprocedure showed near complete resolution of the chemosis with no residual symptoms.

## Figures and Tables

**Figure 1:**
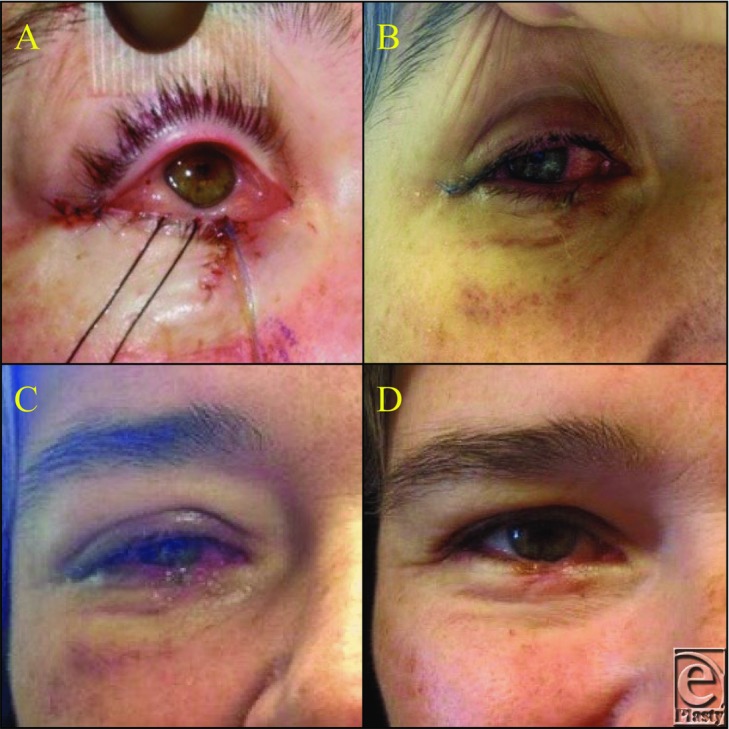
Evolution of intraopertive chemosis. (a) Intraoperative chemosis with obvious conjunctival edema. (b) One week postoperatively with continued evidence of conjunctival irritation. (c) Two weeks postoperatively with resolving conjunctival edema, yet sustained periorbital edema. (d) Six weeks postoperatively with complete resolution of chemosis and edema.
